# Application of the random forest algorithm to *Streptococcus pyogenes* response regulator allele variation: from machine learning to evolutionary models

**DOI:** 10.1038/s41598-021-91941-6

**Published:** 2021-06-16

**Authors:** Sean J. Buckley, Robert J. Harvey, Zack Shan

**Affiliations:** 1 School of Health and Behavioural Sciences, University of the Sunshine Coast, Locked Bag 4, Maroochydore DC, QLD 4558 Australia; 2 Sunshine Coast Health Institute, Birtinya, QLD 4575 Australia; 3 Thompson Institute, University of the Sunshine Coast, Birtinya, QLD 4575 Australia

**Keywords:** Predictive markers, Data processing, Gene regulation, Prokaryote, Genome informatics, Evolutionary genetics, Scientific data

## Abstract

Group A *Streptococcus* (GAS) is a globally significant bacterial pathogen. The GAS genotyping gold standard characterises the nucleotide variation of *emm*, which encodes a surface-exposed protein that is recombinogenic and under immune-based selection pressure. Within a supervised learning methodology, we tested three random forest (RF) algorithms (Guided, Ordinary, and Regularized) and 53 GAS response regulator (RR) allele types to infer six genomic traits (*emm*-type, *emm*-subtype, tissue and country of sample, clinical outcomes, and isolate invasiveness). The Guided, Ordinary, and Regularized RF classifiers inferred the *emm*-type with accuracies of 96.7%, 95.7%, and 95.2%, using ten, three, and four RR alleles in the feature set, respectively. Notably, we inferred the *emm*-type with 93.7% accuracy using only *mga2* and *lrp*. We demonstrated a utility for inferring *emm*-subtype (89.9%), country (88.6%), invasiveness (84.7%), but not clinical (56.9%), or tissue (56.4%), which is consistent with the complexity of GAS pathophysiology. We identified a novel cell wall-spanning domain (SF5), and proposed evolutionary pathways depicting the ‘contrariwise’ and ‘likewise’ chimeric deletion-fusion of *emm* and *enn*. We identified an intermediate strain, which provides evidence of the time-dependent excision of *mga* regulon genes. Overall, our workflow advances the understanding of the GAS *mga* regulon and its plasticity.

## Introduction

*Streptococcus pyogenes* (group A *Streptococcus*: GAS) is a globally significant bacterial pathogen of humans that is responsible for over a half a million deaths worldwide each year^1^. GAS is capable of expressing an arsenal of virulence genes as it survives and thrives in the diverse range of human tissues encountered throughout infection^2^. Distinct from many other bacteria that engage multiple RNA polymerase sigma factors, the growth-phase gene expression of GAS is modulated globally by transcription response regulators (RRs)^3–6^. GAS RRs control factors that mediate metabolism, colonization of tissues, evasion of immunity, stressor response, dissemination, and persistence, by responding to changes in the external and cytosolic microenvironments^7^.

GAS *mga* is the autoregulating RR that controls the expression of genes within the *mga* regulon^8^. The *mga* regulon is bounded by the conserved genes *mga* (*mga1* or *mga2*) and *scpA.* Between *mga* and *scpA* variably lie the highly recombinogenic genes *mrp*, *emm*, and *enn* (encoding the surface-exposed M-related, M-, and M-like proteins)^9^. The canon describes a mosaic of four divergent, yet conserved, cell wall-spanning domains at the 3′ end of *mrp*, *emm*, and *enn*^9,10^. Along with five configurations of *mrp*, *emm*, and *enn*, these cell-wall spanning domains form the basis of the *emm*-pattern typing system^9^. The pattern types consist of A-C ‘throat specialists’ isolates, D ‘skin specialists’, and E ‘generalists’^9^. In 2018, DebRoy and coworkers^11^ observed the chimerisation of the 5′ end of *emm*4 and the 3′ end of its adjacent *enn,* resulting in emm4^C^. This observation defined a novel *emm*-pattern. They established that in 2018, 80% of circulating *emm*4 GAS strains in the United States of America and England contained the *emm*4^C^ variant, highlighting the clinical relevance of this variant.

Phylogenetic delineation by molecular genotyping is central to the understanding of the biology, pathophysiology, epidemiology, and outbreak investigation of bacteria. The gold standard of GAS molecular typing, *emm*-typing, is based on the nucleotide (nt) sequence variability at the 5′ end of *emm*, and specifically the first 30 codons of the mature M-protein^12^. Moreover, *emm*-subtyping provides a higher resolution than *emm*-typing and is based on the nucleotide sequence spanning the C-terminus of the signal sequence (10 amino acids), and the N-terminus of mature M-protein (50 amino acids) (https://www.cdc.gov/streplab/groupa-strep/emm-background.html). *emm*-cluster typing, which is based on four bioinformatic criteria and the phylogeny of the amino acid sequence of the surface-exposed portion of the M-protein, corresponds to human serum binding of the M-protein^13^.

Although *emm*-typing is utilised in nearly all contemporary GAS epidemiological studies^9^, it is well understood that the surface-exposed M-protein is under strong diversifying selection pressure from host immunity^14,15^. Furthermore, the horizontal transfer of *emm* between GAS strains has long been known^14–16^, and more recently termed ‘*emm*-switching’^17,18^. Other studies have also described deletion-fusion (chimeric) events spanning the *emm* locus^11,19–21^. This imposes limitations on the use of *emm*-typing in GAS strain definition. Another popular GAS typing system is the multilocus sequence typing (MLST) system which utilises variation in the nucleotide sequence at the locus of seven genomically-dispersed housekeeping genes, and is often used to augment *emm*-typing^14^. While the *emm*-type/MLST-type pair has higher resolution than either individually, there are many *emm*-types that are represented in multiple MLST-types, and vice versa, which again imposes typing limitations. All of the typing systems described above are amenable to whole-genome sequencing (WGS) approaches.

In the era of WGS, we are increasingly creating more data than can be meaningfully interrogated, meaning that new approaches that enhance the speed and accuracy of data analysis are required. In this context, machine learning (ML) is becoming both ubiquitous and crucial in the subdisciplines of biology, where the growth and centralization of data is exploding^22,23^. What is ML? In general, traditional computer software programming applies rules to data to infer an answer. By contrast, ML flips this paradigm by applying algorithms and statistical analytics to the data and the answer (when available) to infer predictive models^24^. The answer is alternatively referred to as a label. Supervised learning is a ML methodology that can be applied if the label is available, where a model is trained to predict the label (that is, to ‘learn by example’)^25^. Once validated, the model can be applied to test datasets to predict the ‘label’ for unseen datasets. While the popularity of applying ML in biology is rising rapidly, a great concern on the ‘Blackbox-ness’ of the model remains^26^, that is, a biologically interpretable model is preferred to contribute to our understanding of biology. The random forest (RF) is a supervised ML algorithm, that is based on an ensemble of decision trees^27,28^. Each tree is constructed from a random set of input (or predictor) features, and the output of the ensemble is a majority vote of the trees that reduces the risk of inference error introduced by individual trees^29,30^. The RF has several attributes that make it particularly suitable to this study including: robustness, scalability, and its ability to handle both categorical and continuous data types. More importantly, RF generates ML models with a high interpretability.

We hypothesised that the RF algorithm could be applied to the variation in the DNA sequences of GAS RRs to infer genomic traits. We tested this by inferring the *emm*-type, *emm*-subtype, country of origin, preferred tissue of infection, propensity to cause invasive disease, and clinical outcome of GAS isolates using the Ordinary, Regularized, and Guided random forest algorithms.

## Results

### Application of random forest classifiers to infer group A *Streptococcus emm*-type from variation in the response regulator allele types

The accuracy with which the *emm*-type of an isolate was inferred from the 53 selected RR allele types using the three RF classifiers tested ranged from 95.2 to 96.7% (Table 1). The highest and lowest accuracies were observed using the Guided, and Regularized RF classifiers, respectively. The mean accuracy of the three classifiers was 95.9%. The multiclass classification performance metrics including F1, Precision, and Recall are included in Supplementary Table S1.

**Table 1:** Summary of the highest accuracy with which the *emm*-type was inferred when the three tested random forest algorithms were applied to the optimal set of response regulator allele types of group A *Streptococcus*. Predictions were made using tenfold cross validation and 10 replicates.

Random forest algorithm^a^	Accuracy (%)	AUC^b^ (%)	F1^c^ (%)	Precision^c^ (%)	Recall^c^ (%)
Ordinary	95.7	99.8	96.4	94.4	87.4
Regularized	95.2	99.4	97.0	94.7	91.8
Guided	96.7	99.9	97.6	97.0	92.3

^a^The optimal sets for the Ordinary, Regularized, and Guided random forests were [*mga2*, *lrp*, and *gntR_spy0715*], [*mga2*, *lrp*, *copY*, and *crgR*], and [*mga2*, *lrp*, *spy1934*, *gntR_spy0715*, *rivR*, *M28_spy1337*, *spy1325*, *gntR_spy1602*, *spy1817*, and *crgR*], respectively.

^b^AUC = Multiclass classification area under the receiver operating characteristic curve.

^c^Division by zero errors have been excluded from this average.

**Figure 1:**
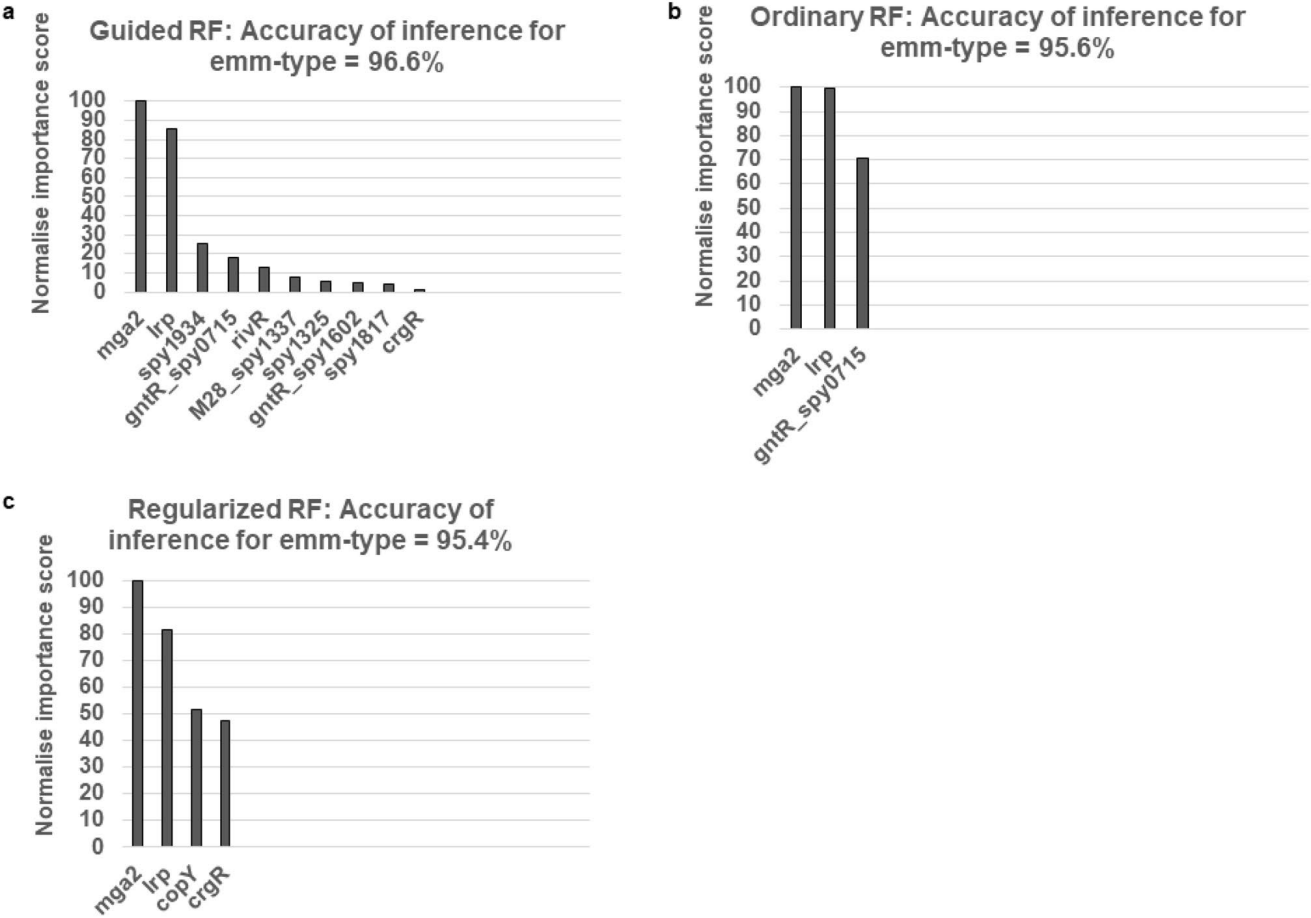
Normalised importance scores of group A *Streptococcus* response regulator (RR) alleles displaying the highest accuracy in inferring the isolate *emm*-type for the three RF classifiers tested. The Guided (**a**), Ordinary (**b**), and Regularized (**c**) RF classifiers employed ten, three, and four RR alleles to attain 96.7%, 95.7%, and 95.2%, respectively. The SPY locus numbers refer to the SF370 isolate, unless stated otherwise.

Figure 1 summarises the normalised non-zero importance scores of the predictor features (RR alleles) selected by each of the RF classifier types in attaining the highest accuracy when inferring the *emm*-type. The Guided (A), Ordinary (B), and Regularized (C) RF classifiers selected ten, three, and four RR alleles to attain 96.7%, 95.7%, and 95.2%, respectively.

The importance score rankings of the minimum feature set of RR alleles required to attain the highest accuracy (optimal feature sets), for inferring the *emm*-type of the three RF classifiers tested are detailed in Table 2. We discovered that each of the RF classifiers had a different number of features in the optimal set. Notably, *mga2* and *lrp* were rank most important in all three. To test the prediction power of *mga2* and *lrp*, we applied the Ordinary, Regular, and Guided classifiers to an input dataset composed of only these two allele types, and were able to predict the *emm*-type with accuracies of 86.7%, 93.7%, and 86.7%, respectively. The mean value was 89.0%.

**Table 2:** Importance value rankings of response regulators alleles (predictor features) in the optimal feature sets inferring GAS *emm*-type for the random forest algorithms tested.

Response regulator	Guided (96.7%)^a^	Ordinary (95.7%)^a^	Regularized (95.2%)^a^
*mga2*	1	1	1
*lrp*	2	2	2
*spy1934*	3		
*spy0715* (*gntR*-like)	4	3	
*rivR*	5		
*M28_spy1337*	6		
*spy1325*	7		
*spy1602* (*gntR*-like)	8		
*spy1817*	9		
*crgR*	10		4
*copY*			3

The optimal feature set is the set of features (from 53 response regulator alleles) selected in attaining the highest accuracy of inferring the *emm*-type for a particular random forest algorithm.

^a^The percentage in brackets is the accuracy of inference.

**Figure 2:**
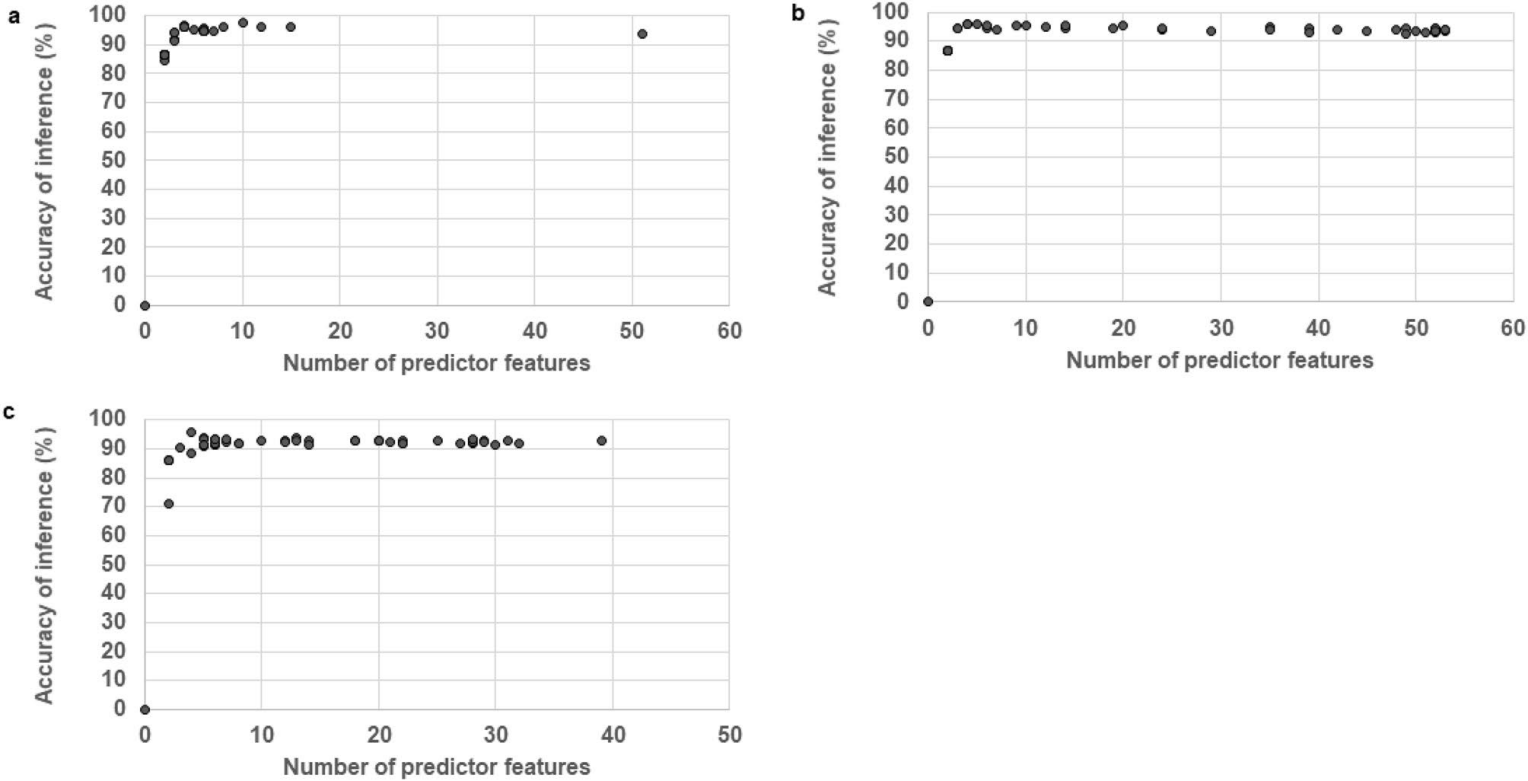
Susceptibility tests. The accuracy of inferring the group A *Streptococcus emm*-type by applying a different number of predictor features (response regulator alleles) to each of the three tested random forest classifiers [(**a**) Guided, (**b**) Ordinary, and (**c**) Regularized).

The susceptibility testing (Fig. 2) shows the relationship between the accuracy of inferring the *emm*-type and the number of predictor features selected for each of the RF classifiers. The curve of best fit for each of the RF classifiers displayed a clear elbow and a minimum threshold number of features below which there was a decline in accuracy of inference with decreasing number of predictor features. While above this threshold the accuracy of inference displayed a relative insusceptible to number of predictor features.

Considering the Guided RF of the highest accuracy of inference, Table 3 lists all of the isolates for which the inferred *emm*-type differed from the observed (published) *emm*-type, and summarises our attempt to assign a putative biological or bioinformatic explanation for the inaccuracy. We identified explanations for ten isolates, which included the following: Prior to testing, it was known that the variation between the RR alleles tested was not able to discriminate *emm*79 from *emm*183, or *emm*101 from *emm*205 (non-discriminatory). Moreover, inference of *emm*-types that only had one representative isolate in the dataset (singletons) was potentially problematic given the methodology used. Similarly inferring the *emm*-type of an isolate that has undergone *emm*-switching or a chimeric *emm*-*enn* event had potential to give inaccurate inferences.

**Table 3:** Examples of inaccurately inferred GAS *emm*-type using the most accurate Guided random forest algorithm and the optimal set of response regulator (RR) allele types.

Strain	*emm*-type^a^		Putative explanations for inaccuracy		
	Observed^b^	Inferred	Non-discriminatory^c^	Singletons^d^	Chimeric *emm*-*enn* event or *emm*-switch^e^
K17011	79 (E3)	183 (E3)	Yes		
K23685	79 (E3)	183 (E3)	Yes		
33181V4T1	205 (E5)	101 (D4)	Yes	*emm*205	
K9612	99 (E6)	182 (E6)		*emm*182	
NGAS148	New type (NT)	5 (M5)		New type^21^	
K23182	63 (E6)	4 (E1)			^11^
K5690	81 (E6)	82 (E3)			^19,21^
NGAS473	82 (E3)	74 (M74)			^19,21^^f^
31140V1S1	98 (D4)	9 (E3)			^21^
33181V1T1_01	137 (E5)	39 (A-C4)			This study
K29655	53 (D4)	52 (D4)			
33123V2S1	71 (D2)	70 (D4)			
K47020	80 (D4)	81 (E6)			
K20641	80 (D4)	81 (E6)			
K33951	80 (D4)	81 (E6)			
20027V1I1	110 (E2)	109 (E4)			
K17074	218 (M218)	119 (D4)			
K9927	223 (D4)	22 (E4)			
K37741	239 (A-C3)	STG866 (NT)			

^a^*emm*-cluster type in brackets.

^b^The observed or published *emm*-type.

^c^Prior to the random forest testing, it was known that the variation between the RR alleles in the feature set was not able to discriminate *emm*79 from *emm*183, or *emm*101 from *emm*205.

^d^Singleton denotes where the dataset contained only one representative of this *emm*-type.

^e^Chimeric *emm*-*enn* events have been observed in isolates of this *emm*-type.

^f^*emm*-switching has also been inferred in this isolate.

### Novel chimeric cell wall-spanning domain and chimeric *emm*-*enn* events

**Figure 3:**
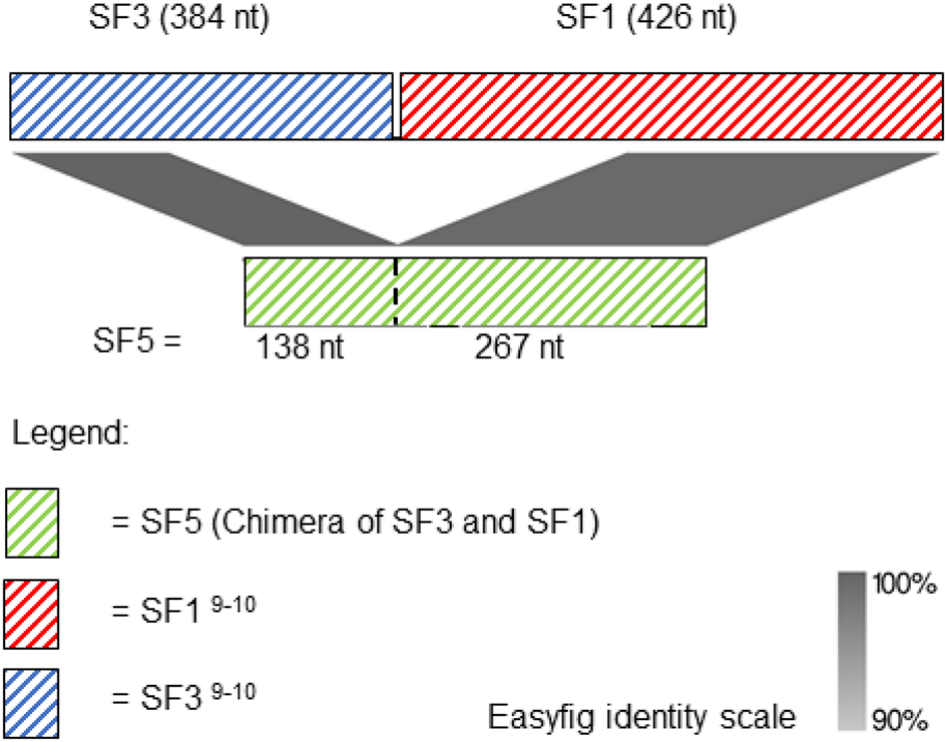
Novel cell wall-spanning domain of group A *Streptococcus* (GAS) *emm*, SF5, described by the chimerisation of SF3 and SF1^9,10^. SF3 and SF1 are typical encoded in the majority of *enn* and a subset of *emm*, respectively. SF5 was observed in *emm*39.4 GAS (31005V6S1) and *emm*137.0 GAS (33181V1T1_01).

We observed a novel cell wall-spanning domain that is described by the chimerisation of SF3 and SF1^9,10^, that we have labelled SF5 (Fig. 3). The nucleotide sequences at the 3′ end of a gene in the *mga* regulon were observed to share 100% identity with SF5 in *emm*39.4 (n = 13 of 13) and *emm*137.0 (n = 2 of 2) isolates.

**Figure 4:**
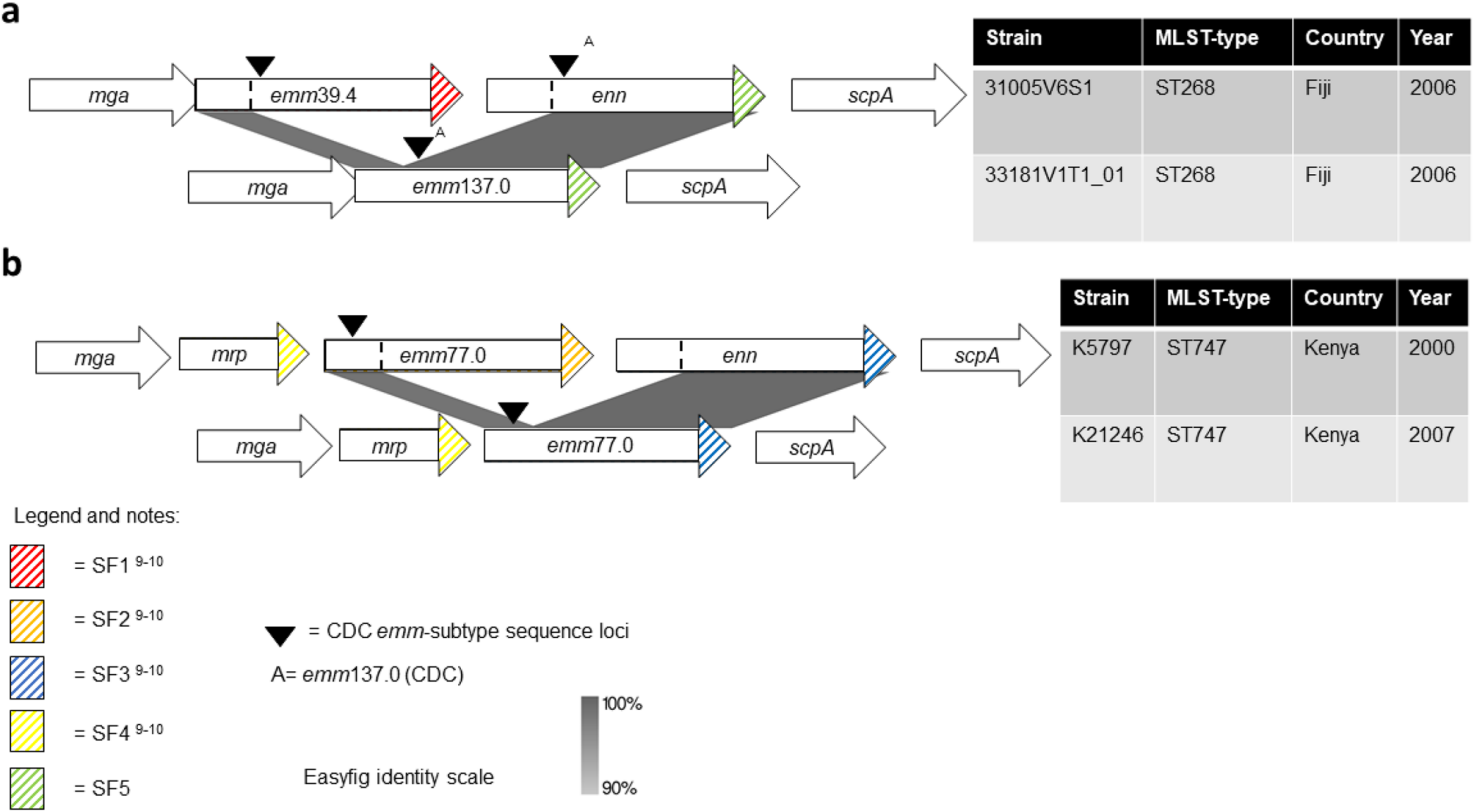
Evolutionary pathways of two novel chimeric *emm*-*enn* events in the *mga* regulon of GAS. (**a**) ‘Contrariwise’ and (**b**) ‘Likewise’ events are depicted were the mutated isolate changes its *emm*-subtype, and retains its *emm*-subtype, respectively. The chimeric *emm*-*enn* is represented by a deletion-fusion event that culminates in a new gene containing the 5′ end of *emm* and the 3′ end of *enn*.

We also observed two novel chimeric *emm*-*enn* events in the *mga* regulon whose evolutionary pathways are depicted in Fig. 4. Note that the Centre for Disease Control and Prevention (CDC) *emm*-subtyping sequence loci of the parental strains (31005V6S1 and K5797) were deleted and retained in the mutant strain, respectively.

While searching for other strains that contained the CDC *emm*137.0 sequence, we noted the following in the draft genome of the Kenya isolate *emm*39.4 ST236 K13190. The largest scaffold (that is, the genome) encoded a contiguous and intact *mga* regulon (*mga*, *emm*, and *scpA*), while a much smaller scaffold (1445 bp) encoded the 3′ end of an SF1-containing *emm* which was adjacent to the 5′ end of *enn* (which contained the 180nt CDC *emm*137.0 sequence). All of which warns that for accuracy, the WGS *emm*-subtype sequences must be read in the context of their position in the *mga* regulon.

### Plasticity in the *mga* regulon of the E3 *emm*-cluster type isolates

**Figure 5:**
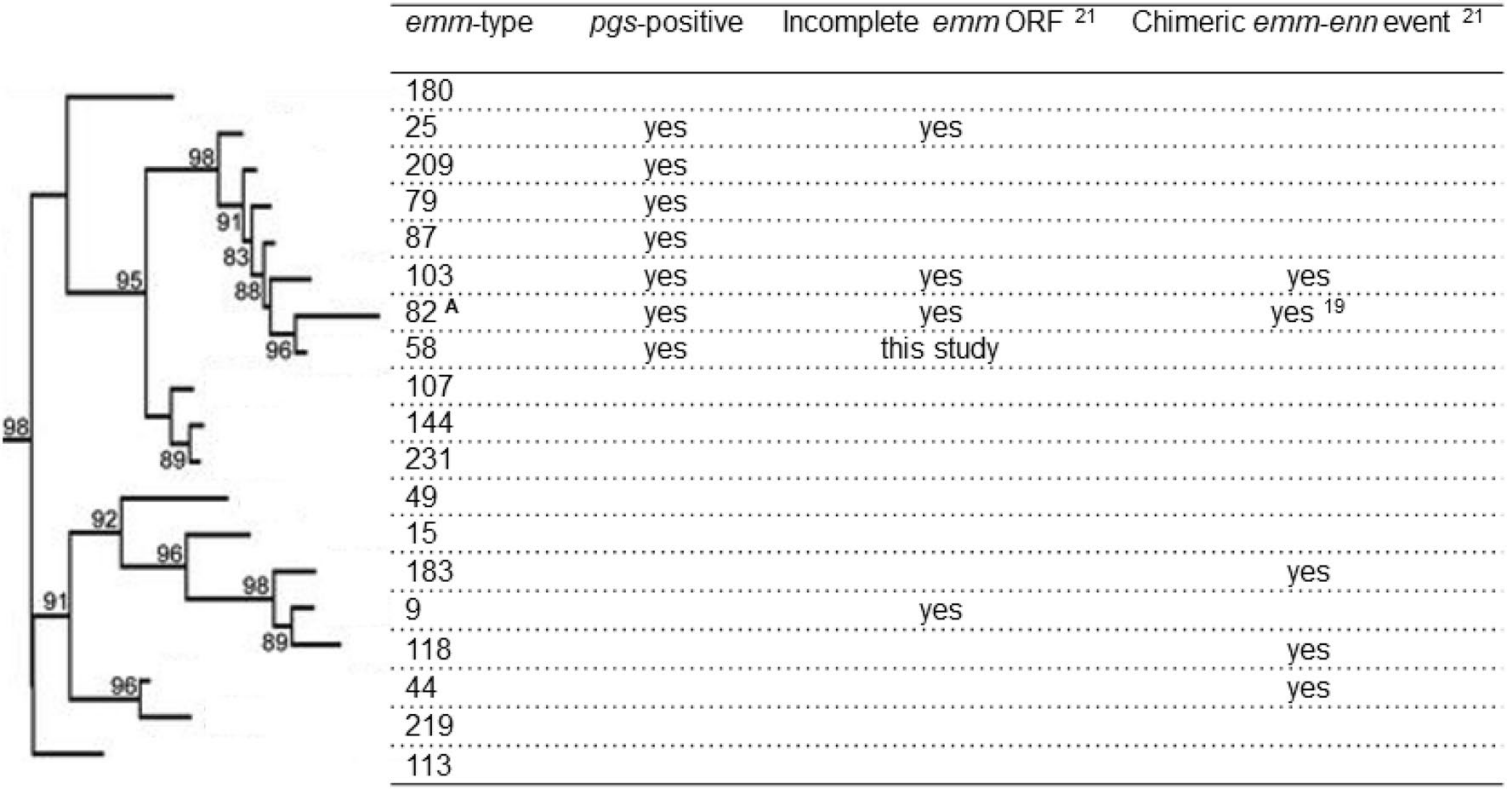
Phylogeny of group A *Streptococcus* E3 *emm*-cluster types. The tree has been labelled with the corresponding *emm*-type. The table summarises examples of recombination and mutation observed in the *mga* regulon of E3-type isolates. The tree is drawn to scale, with branch lengths in the same units (number of amino acid substitutions per site) as those of the evolutionary distances used for the phylogenetic tree. Approximate likelihood-ratio test values > 80% are indicated at the nodes. Adapted from Ref.^13^. Legend: ANGAS473, an emm82 isolate, inferred to have been the result of an emm-switch event has been previously described^18,19^, and was observed in this study to be pgs-negative.

Frost et al.^21^ observed five novel chimeric *emm*-*enn* genes in *emm*9, *emm*44, *emm*58, *emm*73, and *emm*82 isolates. We noted that only *emm*73 is not of *emm*-cluster type E3. Furthermore, Frost’s study revealed 20 incomplete *emm* open reading frames (ORFs), of which the E3 *emm*-cluster type isolates were of *emm*103 (n = 2), *emm*25 (n = 2), *emm*58 (n = 1), *emm*82 (n = 1), and *emm*9 (n = 2) type. Frost and coworkers also observed that *pgs*, encoding a conserved protein between *emm* and *enn*, showed relatively high levels of expression compared to the other *mga* regulon genes, and was only present in E3 *emm*-cluster type isolates. With one exception, we have noted that *pgs* was encoded in all isolates of the monophyletic E3 *emm*-cluster subclade composed of *emm*25, emm58, *emm*79, *emm*82, *emm*87, *emm*103, and *emm*209 types (Fig. 5). This exception was *emm*82 NGAS473 whose *emm*-switch event we have described previously^19^.

**Figure 6:**
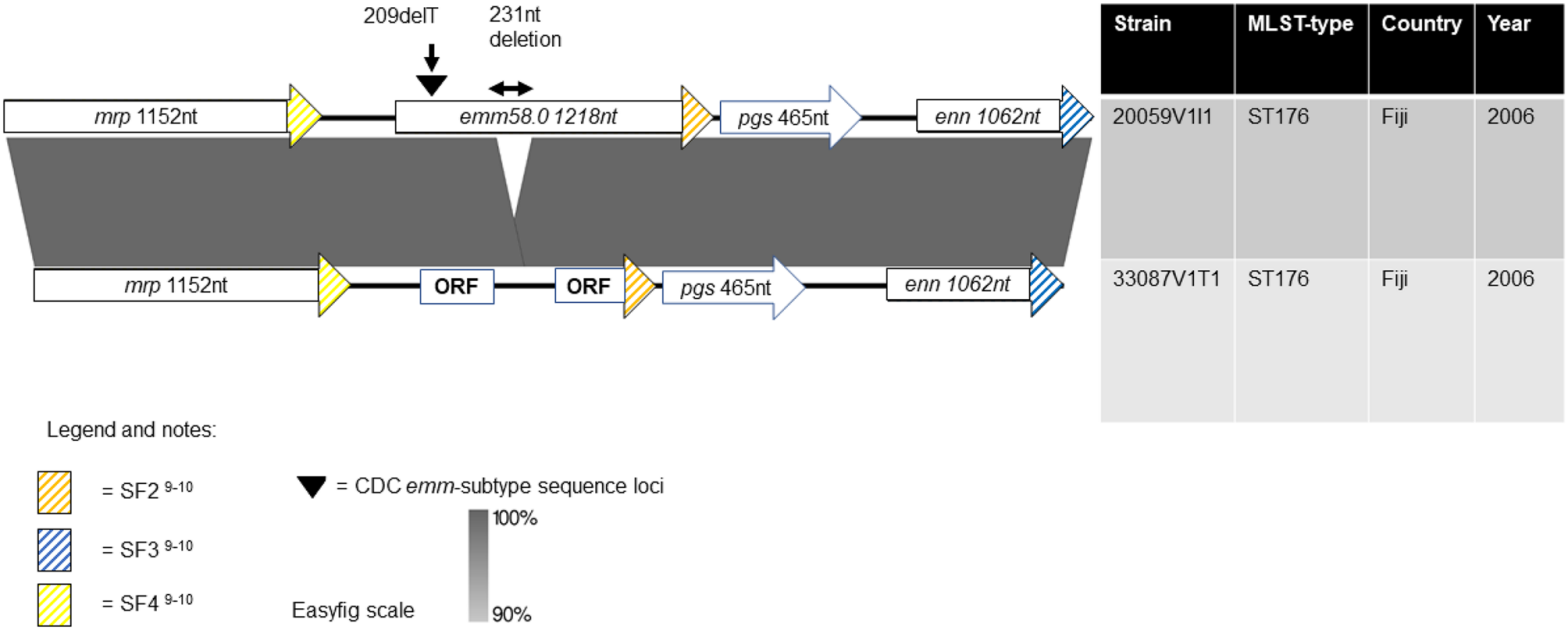
Evolutionary pathway explaining the major disruption to *emm* of group A *Streptococcus* 33087V1T1. This also represents a mechanism for the time-dependent excision of the genes of the *mga* regulon seen in chimeric *emm*-*enn* events. It is likely that the nucleotide deletions observed in 20059V1I1 cause disruption that drastically diminishes the function of *emm,* leading to its eventual deletion.

We observed that *mrp* to *enn* of the Fiji isolate *emm*58.0 ST176 20059V1I1 shared 100% nucleotide identity with the Fiji isolate ST176 33087V1T1 except for a thymine deletion in the CDC *emm*-subtyping sequence (209delT), and a 231 nt deletion starting at the 585th nucleotide of 20059V1I1. The 209delT deletion caused a frameshift and subsequent premature stop codon in *emm*. We therefore propose an evolutionary pathway from strain 20059V1I1 to 33087V1T1 (Fig. 6). Furthermore, it is probable that these deletions have dramatically altered or halted the known function of *emm*. Additionally, the thymine deletion renders the isolate non-typable by the conventional CDC *emm*-subtyping sequence (180nt). We contend that 33087V1T1 could be an intermediate strain that demonstrates a mechanism for the time-dependent excision of genes in the *mga* regulon as seen in chimeric *emm*-*enn* deletion fusion events. Taken together, this provides evidence of the extreme plasticity of the E3 *mga* regulon. In another noteworthy yet non-E3-related observation that highlights the plasticity of the GAS *mga* regulon, we saw that the genome of the Fiji isolate, ST129 *emm*65.4 (33124V1T1) of *emm*-cluster type E6, encoded two fully formed *emm* genes.

### Application of random forest classifiers to infer other group A *Streptococcus* genomic traits from variation in the response regulator allele types

**Figure 7:**
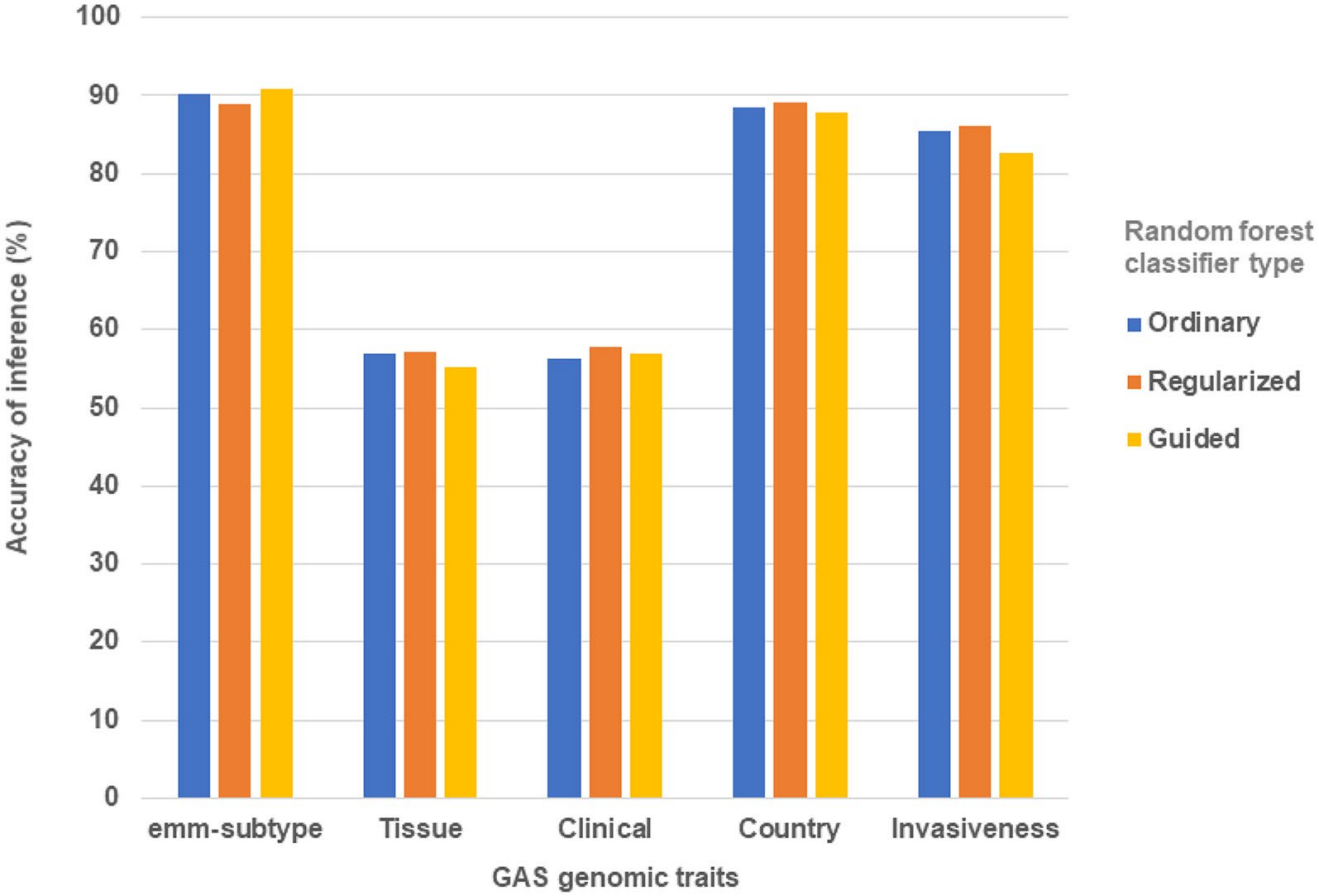
Accuracy of the random forest classifiers tested in inferring group A *Streptococcus* genomic traits from a selection of 53 response regulator allele types. The labels tested include *emm*-subtype, the tissue and country from which the isolate was sampled, clinical outcomes from the infection, and the propensity of the isolate to cause invasive disease.

Figure 7 summarises the accuracy when inferring the other GAS genome traits (*emm*-subtype, tissue, clinical, country, and invasiveness) from variation in the 53 RR alleles using the three RF classifiers tested. Across the three RF classifier types the mean accuracy of inferring the *emm*-subtype, country, invasiveness, clinical, and tissue was 89.9%, 88.6%, 84.7%, 56.9%, and 56.4%, respectively. All of these values were less than the equivalent mean value for the prediction of the *emm*-type (95.9%). Using only *mga2* and *lrp* as the input dataset, we inferred the genomic traits of *emm*-subtype, invasiveness, country, clinical, and tissue of isolates. The mean accuracies of inference of these genomic traits for the three RF classifiers were 84.2%, 83.4%, 83.1%, 53.5%, and 52.4%, respectively. Again, all of these values were less than the equivalent mean value for the prediction of the *emm*-type (89.0%). Along with *emm*-type, these results suggest a potential utility for inferring *emm*-subtype, country, and invasiveness, but not for tissue and clinical. This last observation is consistent with the complexity of the interaction between the pathogenic GAS isolate and the immune system of the infected host.

## Discussion

In this study we applied three RF algorithms to the variation in a selection of GAS RR allele types in order to infer the *emm*-type of the isolate with high accuracy. This analysis enabled us to infer the *emm*-subtype, country of sample, and invasiveness of the isolate. However, we were not able to accurately infer the tissue sampled or clinical outcomes of the infection. We investigated the causes of inaccuracy when inferring the *emm*-type using the optimal Guided RF feature set because it was the most accurate configuration for this purpose. From this we identified a novel chimera of the conserved cell wall spanning domains, SF3 and SF1^9,10^, that we have labelled SF5 (Fig. 3). We also identified two novel chimeric *emm*-*enn* events in the *mga* regulon. These events were in *emm*77.0 and *emm*39.4 type isolates. We defined the events seen in the *emm*77.0 and *emm*137.0 isolates as ‘likewise’ (Fig. 4b) and ‘contrariwise’ (Fig. 4a), respectively. Finally, we proposed an evolutionary pathway describing the disrupted *emm* of the E3-type Fiji strain 33087V1T1, from which we contend that this isolate represents an intermediate strain that suggests a mechanism for the time-dependent excision of genes in the *mga* regulon.

### Application of the random forest algorithm and the response regulator allele types to infer the *emm*-type

We have demonstrated that using each of the three types of RF tested we were able to infer the GAS *emm*-type from the variation in the selected feature sets of 53 RR allele types with high accuracy. This is important because it represents an alternative to the *emm*-based systems whose accuracy is susceptible to the plasticity of the *mga* regulon. Additionally, it shows that RR-based typing is backwards compatible with the vast *emm*-centric GAS knowledge base. Table 4 collates the relative strengths and weaknesses of the *emm*-based and RR-based typing system.

**Table 4:** Comparison of properties of the *emm*-based and response regulator-based typing systems of group A *Streptococcus*.

*emm*-based typing	Response regulator allele-based typing
*emm* is a surface exposed protein	The RRs are a family of cytosolic proteins that share broadly similar functional domains, including control of the expression of traditional GAS typing proteins
*Known to be antigenic*	**Not known to be antigenic**
*Under strong positive selection pressure from host immunity*^16^	**Many proteins that are primarily under negative selection pressure**^19^
*Single locus (or single point of failure)*	**Multiple genomically-dispersed loci (redundancy)**
*Highly recombinogenic locus*	Many proteins with a range of recombinogenicity
**Genotype-dependent technique**	**Genotype-dependent technique**
**Amenable to WGS-derived techniques**	**Amenable to WGS-derived techniques**
**Vast *emm*-centric knowledge base**	**Backwards compatible with *emm* knowledge base (this study)**
*Has limitations in identifying and explaining rare mga regulon anomalies*	**Shows potential as a ‘cross reference’ in identifying and explaining rare *mga* regulon anomalies (this study)**

Preferred (bold) and non-preferred (italics) properties of a molecular bacterial typing system.

We were able to describe the feature sets (of RRs) that attained the highest accuracy of predicting *emm*-type (that is, optimal feature sets) for each of the RF algorithms (Table 2). The highest overall accuracy was attained using the Guided RF with the following ten RRs: *mga2*, *lrp*, *spy1934*, *spy0715* (*gntR*-like), *rivR*, *M28_spy1337*, *spy1325*, *spy1602* (*gntR*-like), *spy1817*, and *crgR*. The optimal feature set for the RF algorithms were of different composition (that is, size and constituents).

It should be noted that *mga2* and *lrp* were ranked most important in all three, suggesting a mathematical importance in inferring *emm*-type. Of the 53 RR allele types tested, *mga2* had the highest ranking importance score when inferring *emm*-type for all three RF algorithms. The following are offered as reasons why *mga2* ranked highest. Biologically, *mga* is an important response regulator which controls the expression of more than 10% of the GAS genome, and Mga is a large enough protein (62 kDa) to contain multiple functional domains^31^. Furthermore, *mga* is encoded proximally to *emm*, and regulates the transcription of *emm*. Mathematically, we also have previously measured that *mga2* had the highest number of unique allele types of 35 of the 53 RRs tested in this dataset^19^. Thus, biologically and mathematically, it is predicable that the importance score of *mga2* would rank highly when inferring the *emm*-type.

We also saw a threshold number of feature variables (RRs) below which the accuracy declined, but above which the accuracy showed a relative insusceptibility to the number of predictor variables (RRs) (Fig. 2). This is of different significance for both in silico and laboratory-based analyses. While the impost of testing all 53 RRs in silico is negligible, a reduction to ten variables (RRs) may represent a significant economic saving of resources in the laboratory. Furthermore, reduction of predictor variables (RRs) in this in silico, WGS-based analysis was predicated on an increase in accuracy. Thus, in vitro testing must consider the trade-off of accuracy when decreasing the number of RRs tested on the basis of economics. Regardless, our findings represent a significant reduction in the search space and a logical starting test set for in vitro studies.

We endeavoured to understand when our process inferred the wrong *emm*-type for the Guided RF. That is, the inferred and published *emm*-type were different. We were able to propose putative biological explanations for approximately half of examples of inaccurate inferences (Table 3). These included the following scenarios. Firstly, isolates of either *emm*105 or *emm*205 type that shared identical RR allele types but have differing *emm*-types were at risk of incorrect typing. Similarly, the variation in the RR alleles of the optimal feature set of the Guided RF could not discriminate *emm*79 from *emm*183. Secondly, as a by-product of the supervised learning methodology employed, singleton *emm*-types were also at risk of inaccurate inference if that isolate had not been included in the training genome set. Thirdly, the singletons may also include examples of *emm*-switching and chimeric *emm*-*enn* events, although these occur relatively infrequently. Furthermore, inference of *emm*-type in the examples of *emm*-switching is problematic because the background RR allele types are likely to reflect the recipient isolate and not the donor of the recombined *emm*. Similarly, for chimeric *emm*-*enn* events, the background RR alleles will likely reflect that of the pre-event parental isolate which may have had a different *emm*-type. Together these anomalies had the unintended consequence of forming the basis of a preliminary method for identifying *emm*-switching and chimeric *emm*-*enn* events, which is a current unmet need in the GAS community.

### Utility of the random forest in identifying chimeric cell wall-spanning domain and chimeric *emm*-*enn* events

We observed that the ST268 *emm*137.0 isolate (33181V1T1_01), that had been sampled from Fiji in 2006, was incorrectly inferred to be of *emm*-type 39 using the RRs and the Guided RF. Upon closer inspection we identified that 33181V1T1_01 possessed a novel cell wall-spanning domain, SF5, which is a chimerisation of two of the canonical cell wall-spanning domains (SF3 and SF1). SF5 was also observed in the ST268 *emm*39.4 isolate (31005V6S1), which had also been isolated from Fiji in 2006.

Additionally, *emm*137.0 of 33181V1T1_01 shared 100% nucleotide identity with the chimeric fusion of the 5′ end of *emm* and the 3′ end of *enn* of 31005V6S1. We noted that the CDC *emm*-subtyping sequence locus of the parental strain (31005V6S1) was deleted from the mutant strain (33181V1T1_01). We propose that this represents a novel chimeric *emm*-*enn* event (‘contrariwise’), that is visually depicted in Fig. 4a. We also identified that the ST747 *emm*77.0 isolate (K21246), which had been sampled from Kenya in 2007, possessed SF3 at the 3′ end of *emm*, noting that SF3 is canonically encoded in *enn*. The ST747 *emm*77.0 isolate (K5797) had been sampled from Kenya in 2000. We observed that *emm*77.0 of K21246 shares 100% identity with the fusion of the 5′ end of *emm* and the 3′ end of *enn* of K5797, noting that the CDC *emm*-subtyping sequence locus of the parental strain (K5797) was retained in the mutant strain (K21246). We propose that this too represents a novel chimeric *emm*-*enn* event (‘likewise’), that is visually depicted in Fig. 4b. The observed configurations of the conserved cell wall-spanning domains provided additional evidence to support our proposed evolutionary pathways.

We have chosen to define the first of the chimeric *emm*-*enn* events as ‘contrariwise’ (33181V1T1_01) and the second as ‘likewise’ (K21246) in preference to synonymous and non-synonymous to avoid confusion with established molecular biology nomenclature. Furthermore, typing of these mutants should be *emm*137^C^ (33181V1T1_01) and *emm*77^C^ (K21246) for consistency with the prevailing convention for chimeric *emm* and M-like genes^11,19,21^. It should be noted that historically contrariwise events may have been labelled as *emm*-switches. Where the evidence supports, we recommend that the term *emm*-switch is reserved for recombination events that have involved horizontal gene transfer of DNA containing *emm*. The recombinant DNA may also include DNA, that is adjacent to *emm*, other than that encoding *emm*. Recombination events have been identified in *emm*82^C^ isolates, from which it can be inferred that the isolates have undergone both an *emm*-switch and a ‘likewise’ chimeric *emm*-*enn* event^18,19^. Finally, findings in this part of the project confirmed the bioinformatics-related imperative of reading *emm*-subtyping sequences in the context of their location within the *mga* regulon, when typing WGS genomes.

### Plasticity in the *mga* regulon of the E3 *emm*-cluster type isolates

We observed a high degree of disruption to the E3 *mga* regulon caused by mutation and recombination. Additionally, we proposed an evolutionary pathway describing the disrupted *emm* of the E3-type Fiji strain 33087V1T1. Given this disruption we would expect to see either one of two eventualities. Firstly (and unlikely), the disrupted *emm* is retained in the genome because it confers advantage and is selected in the population. Or secondly (and more likely given the pre-eminence of *emm* in host immune evasion), that further deleterious mutations are acquired and the locus is gradually excised. Therein, we contend that this isolate represents an intermediate strain, suggesting a mechanism for the time-dependent excision of genes in the *mga* regulon. This represents an important finding in the evolutionary history of E3 GAS. Our findings are also epidemiologically important, because E3-type isolates have recently been identified in outbreaks as the causative agent of severe GAS disease^18,32–35^. Furthermore, given that *emm* is a major focus in GAS vaccine development, we consider that this work increases our understanding of rare and anomalous *mga* regulons. From a bioinformatics perspective, we contend also that it would not be prudent to assign an *emm*-subtype to the likes of 33087V1T1 (the non-typable intermediate strain evolved from *emm*58.0 20059V1I1) using the *emm* locus when a frameshift has disrupted and likely deleted the function of *emm*, making it susceptible to future excision. Overall, we assert that the plasticity of the E3 *mga* regulon represents a ‘snap shot’ of the real-time evolution of GAS and represents a recombination hotspot.

### Application of the random forest algorithm and the response regulator allele types to infer other GAS genomic traits

A recent review by Allen et al. highlights the extensive opportunity that exists for the application of machine learning in microbiological genomics^36^. In this study, we were able to accurately predict the *emm*-subtype, the country from which the isolate was sampled, and the invasiveness of the isolate. Our work stands as template for predicting other untested GAS genomic traits using RF and RR allele types. Given the accuracy of the inference of invasiveness, we would suggest potential utility in an in vitro assay for predicting invasive GAS isolates.

This process flow was ineffective at inferring the tissue from which the isolate was sampled, and the clinical outcomes. Given the complexity of the GAS-host immunity interaction, this is not an unexpected result. Furthermore, it inspires an exciting question. Can a higher degree of accuracy be achieved if the input data set is augmented with judiciously selected human gene allele types?

## Conclusions

In this study we applied the RF algorithm to the variation in a set of GAS RR alleles and were able to infer the *emm*-type with high accuracy. The highest accuracy, 96.7%, was achieved using the Guided RF. We identified the optimal feature sets (of RRs) for three different RF classifiers, therein describing how many, and which alleles had greatest importance when inferring the *emm*-type with the greatest accuracy. We observed that each RF classifier had a threshold number of features below which the accuracy of inference dropped, but above which the accuracy was relatively insusceptible to the number of features. By examining the potential sources of inaccuracy, we discovered a novel *mga* regulon cell wall-spanning domain, SF5. We also proposed two novel evolutionary pathways of chimeric *emm*-*enn* events in the *mga* regulon in which the original *emm*-type was retained by one, but was changed in the other. We defined these as ‘likewise’ and ‘contrariwise’ events, respectively. We also proposed an evolutionary pathway that describes frameshift mutation-induced disruption to *emm*, which results in a strain that represents an intermediate step in the time-dependent excision of genes in the *mga* regulon. We were also able to usefully predict *emm*-subtype, the country from which the isolate was sampled, and the invasiveness of the isolate. However, we were unable to predict the tissue from which the isolate was sampled, or the clinical outcome from the infection. Thus, ML has allowed us to interpret the biology of GAS and propose new evolutionary models. Noting that the RF has been under-utilised in the GAS community to date, we propose that our process flow serves as a template for the prediction of other untested GAS genomic traits.

## Methods

### Rationale

This study was designed to test whether the nucleotide sequences of GAS RR genes (predictor features) can predict genomic traits (labels). The following summarises our justification for investigating RRs. Traditional studies of GAS virulence have primarily focused on the biofunction of GAS virulence genes (including exotoxins, DNases, proteases, surface-exposed adherence-related proteins, and other bioactive enzymes) to the widespread exclusion of the co-ordinate regulation of the initiation of transcription of the aforementioned virulence genes. GAS RRs regulate the transcription of the majority of GAS virulence genes (and often themselves because many are autoregulating). Furthermore, GAS RRs are the major regulators of the GAS gene expression profile in the growth phase, in lieu of sigma factors. Therein, we hypothesised that variation in DNA sequences of the RR genes might correlate with GAS genomic traits. Regardless, the workflow developed in this study should represent a viable template for the investigation of untested GAS virulence genes in the future.

The RF ML algorithm was chosen because it is robust, scalable, capable of processing large datasets with high dimensionality and heterogeneous feature (or variable) types, and provides high interpretability. The three RFs tested were the Ordinary, Regularized, and Guided^27,37^. The regularized RF introduces a penalty to the inclusion of a new feature during decision tree building. Thus, a regularized RF only adds new features if those new features provide substantial new predictive information. The guided RF uses the importance scores from an ordinary random forest to guide the feature selection. Furthermore, we intended to find the maximum accuracy with which we could infer the response variables (genomic traits), and investigate the susceptibility of inference accuracy to the reduction of predictor feature set size.

### Input data

**Figure 8:**
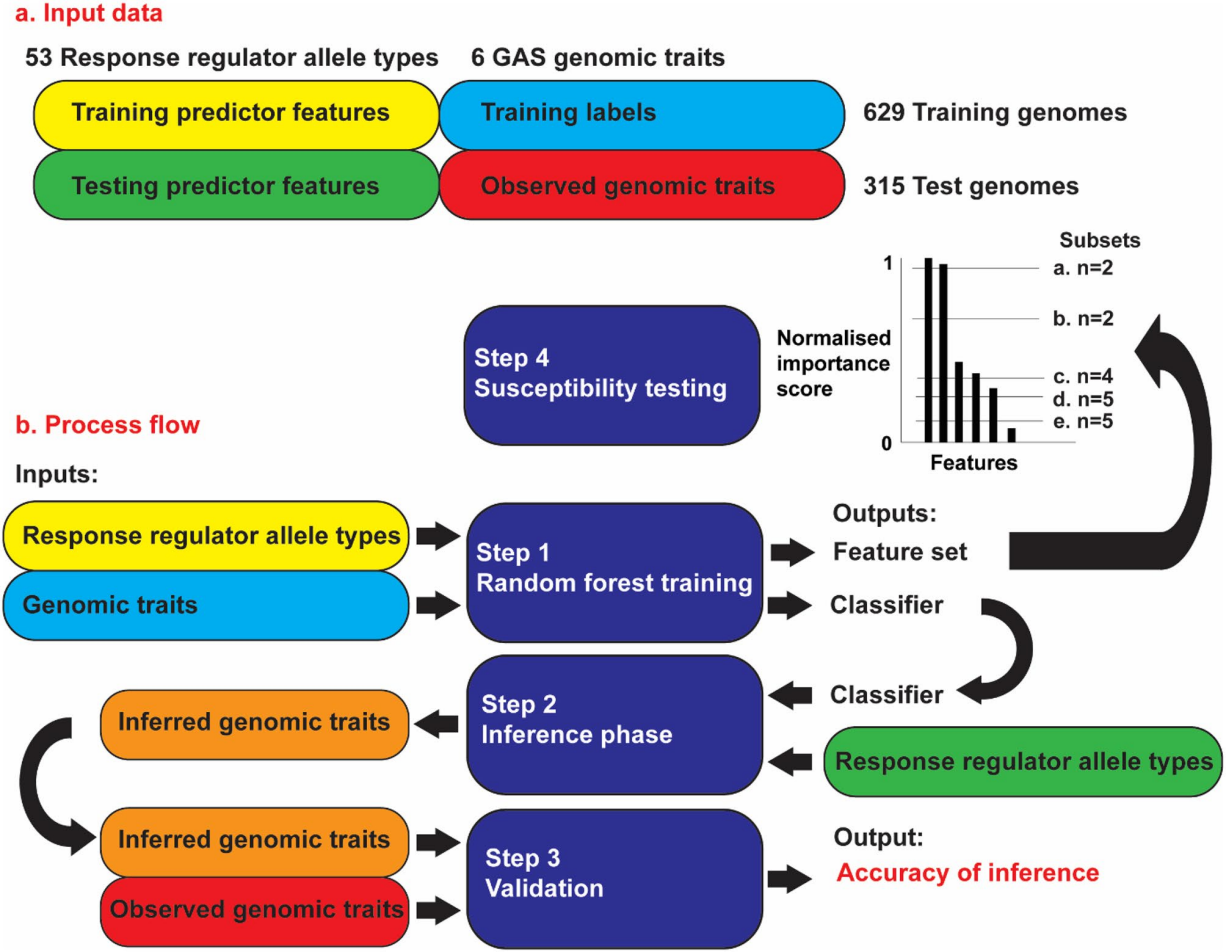
Summary of (**a**) input data nomenclature and (**b**) process flow of this study.

The input data was composed of 53 nucleotide-based GAS RR allele types and six genomic traits, extracted from 944 genomes (Fig. 8a). Of these allele types, 35 have been described previously^19^, and 18 were less well characterised or putative RRs which still displayed the characteristic helix-turn-helix DNA binding domain inferred by SMART domain^38^. The six genomic traits were the *emm*-type, *emm*-subtype, the human tissue (tissue) and country from which the GAS isolate was sampled, clinical outcomes observed from the isolate (clinical), and the propensity for the isolate to cause invasive disease (invasiveness). The genomic traits of tissue and clinical correspond to the metadata fields that are titled ‘tissue/source’ and ‘clinical’ as previously described^19,39^. ‘Tissue/source’ essentially represents the tissue sampled, ‘clinical’ is an assemblage of presentation and disease outcome. The input data is included in Supplementary Table S2. The SPY locus numbers refer to the SF370 isolate, unless otherwise denoted. To minimise possible overfitting, the genomes were randomly separated into the training (n = 629) and test (n = 315) sets. *emm*-types that had only one representative in the dataset (singletons) were included in the training set. Furthermore, prior to testing with the RF algorithms, we established that the dataset contained a pair (n = 2 of 125) of different *emm*-types that shared identical RR allele types (*emm*105 and *emm*205).

### Process flow

Step 1 of this study involved training the RF classifier using the input data of the RR allele types as training predictor features and the genomic traits as training labels (Fig. 8b). Output data generated from this step were the selected predictor feature set importance scores of the RF classifier. In RF feature selection, the redundant or irrelevant features were eliminated under the guidance of the feature importance scores^40^. Simplistically, the feature importance score is a measure of the importance of the attribute in inferring the correct classification^41^. In step 2 the RF classifier was applied to the RR alleles types (test predictor features) to infer the classification of the six genomic traits (labels). The inferred genomic traits were then compared to the observed genomic traits, and an accuracy of inference was calculated in step 3.

**Figure 9:**
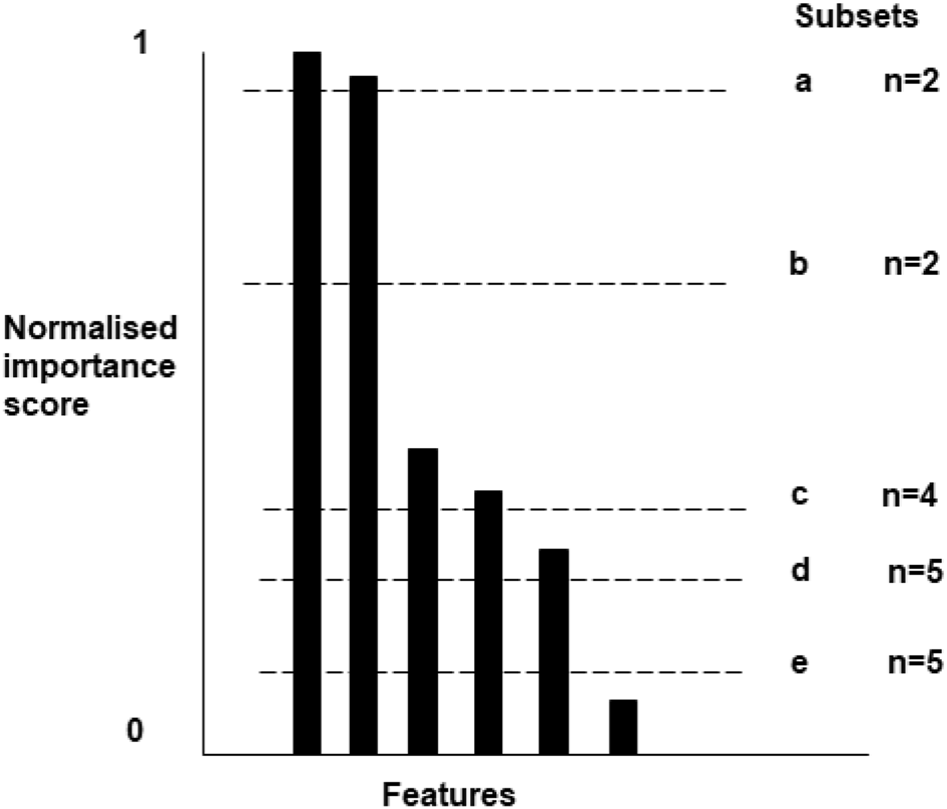
Schematic representation of the normalised importance score plot for the selected predictor feature set. Subsets of features were selected based on arbitrary threshold normalised importance values. Steps 2 and 3 of the process flow were then applied to each of these subsets.

In step 4, susceptibility testing, the histogram of the normalised importance scores of the feature selection set was investigated (Fig. 9). Step 4 was designed to select subsets of predictor features each containing the nth most important features with the intention of maximising the accuracy of inference. A threshold normalised importance score was arbitrarily set at a value close to 1, therein defining a subset of the selected predictor feature set that only contained the features with the highest importance scores. This was repeated for multiple arbitrarily decreasing threshold importance scores, creating subsets with increasingly more features. Steps 2 and 3 were then applied to each of these subsets and plots were generated to assess the susceptibility of the accuracy of inference to the number of predictor features (RR allele types). Steps 1–4 were repeated for the three different RF classifier algorithms.

### Cross validation and multiclass classification performance evaluation for the optimal feature sets

We defined the optimal feature set as the set of response regulators from which the *emm*-type was inferred with the highest accuracy for each of the three random forest algorithms. Ten-fold cross validation with ten replicates was performed to measure the stability of these inferences using the ‘caret’^42^ and ‘RRF’ R-code packages^37^. As previously described, the random forest is an ensemble learning method, in which the classifier is derived from the voting results of multiple decision trees. Inferring *emm*-type from this dataset was a multiclass classification. In order to evaluate the performance of these multiclass classifications, we employed the ‘pROC’ R-code package^43^ to estimate the multiclass area under the receiver operating characteristic curve (AUC)^44^. The multiclass.roc function of ‘pROC’ was applied to predictions determined using the ‘vote’ parameter in the predict function of the ‘RRF’ package. We also collated the F1, Precision, and Recall metrics. The R-code was implemented in version 1.1.447, and is included as Supplementary File S3.

### Development of evolutionary pathway models

In the development of the evolutionary pathways, the chimerisation of genes was confirmed using BLASTn^45^, as implemented in Geneious V 8.1.9^46^, and visually depicted using Easyfig^47^.

## Supplementary Information

Supplementary File 3.

Supplementary Table 1.

Supplementary Table 2.

## References

[1] Sanyahumbi, A. S., Colquhoun, S., Wyber, R. & Carapetis, J. R. Global disease burden of group A *Streptococcus*. *Streptococcus pyogenes: basic biology to clinical manifestations* 2016 Feb 10. In *Streptococcus pyogenes: Basic Biology to Clinical Manifestations* (eds. Ferretti, J. J. *et al.*) (University of Oklahoma Health Sciences Center, 2016). Accessed 6 June 2021. https://www.ncbi.nlm.nih.gov/books/NBK333415/.

[2] Walker MJ (2014). Disease manifestations and pathogenic mechanisms of group A *Streptococcus*. Clin. Microbiol. Rev..

[3] Helmann, J. D., Moran, C. P. RNA polymerase and sigma factors. in *Bacillus subtilis and Its Closest Relatives*. 287–312 (ASM Press, 2014). 10.1128/9781555817992.ch21

[4] Opdyke JA, Scott JR, Moran CP (2001). A secondary RNA polymerase sigma factor from *Streptococcus pyogenes*. Mol. Microbiol..

[5] Beyer-Sehlmeyer G, Kreikemeyer B, Hörster A, Podbielski A (2005). Analysis of the growth phase-associated transcriptome of *Streptococcus pyogenes*. Int. J. Med. Microbiol..

[6] Woodbury RL, Wang X, Moran CP (2006). Sigma X induces competence gene expression in *Streptococcus pyogenes*. Res. Microbiol..

[7] McIver KS (2009). Stand-alone response regulators controlling global virulence networks in *Streptococcus pyogenes*. Bact. Sens. Signal..

[8] McIver KS, Heath AS, Green BD, Scott JR (1995). Specific binding of the activator Mga to promoter sequences of the *emm* and *scpA* genes in the group A *streptococcus*. J. Bacteriol..

[9] Bessen, D. E., Smeesters, P. R. & Beall, B. W. Molecular epidemiology, ecology, and evolution of group a streptococci. Gram-Positive Pathogens. 177–203. 10.1128/9781683670131.ch12 (ASM Press, 2019). 10.1128/microbiolspec.cpp3-0009-2018 PMC11633622 30191802

[10] Hollingshead SK, Arnold J, Readdy TL, Bessen D (1994). Molecular evolution of a multigene family in group A *streptococci*. Mol. Biol. Evol..

[11] DebRoy S (2018). Identification of a chimeric *emm* gene and novel *emm* pattern in currently circulating strains of *emm4* Group A *Streptococcus*. Microb. Genom..

[12] Beall B, Facklam R, Thompson T (1996). Sequencing *emm*-specific PCR products for routine and accurate typing of group A *streptococci*. J. Clin. Microbiol..

[13] Sanderson-Smith M (2014). A systematic and functional classification of *Streptococcus pyogenes* that serves as a new tool for molecular typing and vaccine development. J. Infect. Dis..

[14] Enright MC, Spratt BG, Kalia A, Cross JH, Bessen DE (2001). Multilocus sequence typing of *Streptococcus pyogenes* and the relationships between *emm* type and clone. Infect. Immun..

[15] McGregor KF (2004). Multilocus sequence typing of *Streptococcus pyogenes* representing most known *emm* types and distinctions among subpopulation genetic structures. J. Bacteriol..

[16] Bessen DE, McGregor KF, Whatmore AM (2008). Relationships between *emm* and multilocus sequence types within a global collection of *Streptococcus pyogenes*. BMC Microbiol..

[17] Turner CE (2019). The emergence of successful *Streptococcus pyogenes* lineages through convergent pathways of capsule loss and recombination directing high toxin expression. MBio.

[18] Chochua S (2017). Population and whole genome sequence based characterization of invasive group A *streptococci* recovered in the United States during 2015. MBio.

[19] Buckley SJ, Davies MR, McMillan DJ (2020). In silico characterisation of stand-alone response regulators of *Streptococcus pyogenes*. PLoS ONE.

[20] Athey TB (2014). Deriving group A *Streptococcus* typing information from short-read whole-genome sequencing data. J. Clin. Microbiol..

[21] Frost HR (2020). Analysis of global collection of group A *Streptococcus* genomes reveals that the majority encode a trio of M and M-like proteins. Msphere..

[22] Camacho DM, Collins KM, Powers RK, Costello JC, Collins JJ (2018). Next-generation machine learning for biological networks. Cell.

[23] Ghassemi M (2020). A review of challenges and opportunities in machine learning for health. AMIA Summits Transl. Sci. Proc..

[24] Allison, K. & Moroney, L. Machine learning zero to hero [video file]. Accessed 10 May 2010. https://www.youtube.com/watch?v=VwVg9jCtqaU

[25] Tabell Johnsson, M. & Jafar, A. (2020) (Dissertation). Accessed 6 June 2021. http://urn.kb.se/resolve?urn=urn:nbn:se:bth-20218.

[26] Castelvecchi D (2016). Can we open the black box of AI?. Nat. News.

[27] Breiman L (2001). Random forests. Mach. Learn..

[28] Osisanwo F (2017). Supervised machine learning algorithms: Classification and comparison. Int. J. Comput. Trends Technol. IJCTT.

[29] Speiser JL, Miller ME, Tooze J, Ip E (2019). A comparison of random forest variable selection methods for classification prediction modeling. Expert Syst. Appl..

[30] Rashidi HH, Tran NK, Betts EV, Howell LP, Green R (2019). Artificial intelligence and machine learning in pathology: The present landscape of supervised methods. Acad. Pathol..

[31] Hondorp ER, McIver KS (2007). The Mga virulence regulon: Infection where the grass is greener. Mol. Microbiol..

[32] Campbell PT (2020). Longitudinal analysis of group A *Streptococcus emm* types and *emm* clusters in a high-prevalence setting: Relationship between past and future infections. J. Infect. Dis..

[33] Athey TB (2016). High incidence of invasive group A *Streptococcus* disease caused by strains of uncommon *emm* types in Thunder Bay, Ontario, Canada. J. Clin. Microbiol..

[34] Tyrrell, G. J., Fathima, S., Kakulphimp, J. & Bell, C. In *Open Forum Infectious Diseases.* ofy177 (Oxford University Press US). 10.1093/ofid/ofy177 PMC6084600 30109241

[35] Gherardi G, Vitali LA, Creti R (2018). Prevalent *emm* types among invasive GAS in Europe and North America since year 2000. Front. Public Health.

[36] Allen, J. P., Snitkin, E., Pincus, N. B., Hauser, A. R. Forest and Trees: Exploring Bacterial Virulence with Genome-wide Association Studies and Machine Learning. Trends in Microbiology. 10.1016/j.tim.2020.12.002 (Elsevier BV, 2021). 10.1016/j.tim.2020.12.002 PMC8187264 33455849

[37] Deng, H. Guided random forest in the RRF package. arXiv preprint arXiv::1306.0237 (2013).

[38] Schultz J, Copley RR, Doerks T, Ponting CP, Bork P (2000). SMART: A web-based tool for the study of genetically mobile domains. Nucleic Acids Res..

[39] Davies MR (2019). Atlas of group A *streptococcal* vaccine candidates compiled using large-scale comparative genomics. Nat. Genet..

[40] Deng H, Runger G (2013). Gene selection with guided regularized random forest. Pattern Recogn..

[41] Livingston, F. Implementation of Breiman’s random forest machine learning algorithm. *ECE591Q Mach. Learn. J. Pap.* 1–13 (2005).

[42] Kuhn M (2008). Building predictive models in R using the caret package. J. Stat. Softw..

[43] Robin X (2011). pROC: An open-source package for R and S+ to analyze and compare ROC curves. BMC Bioinform..

[44] Hand DJ, Till RJ (2001). A simple generalisation of the area under the ROC curve for multiple class classification problems. Mach. Learn..

[45] Altschul SF, Gish W, Miller W, Myers EW, Lipman DJ (1990). Basic local alignment search tool. J. Mol. Biol..

[46] Kearse M (2012). Geneious Basic: An integrated and extendable desktop software platform for the organization and analysis of sequence data. Bioinformatics.

[47] Sullivan MJ, Petty NK, Beatson SA (2011). Easyfig: A genome comparison visualizer. Bioinformatics.

