# Supplementary material for: Application of the random forest algorithm to Streptococcus pyogenes response regulator allele variation: from machine learning to evolutionary models
Source: Sci Rep. 2021 Jun 16;11:12687. doi: 10.1038/s41598-021-91941-6 (PMC8209152; doi:10.1038/s41598-021-91941-6)
Supplement: Supplementary file 1 — Supplementary File 3. [file 41598_2021_91941_MOESM1_ESM.pdf]

Supplementary File S3: R-code for cross validation and multiclass classification performance evaluation

```
library(caret)
library(RRF)
library(doSNOW)
library(pROC)

###########################################
# Ordinary random forest: Cross validation
###########################################

all<-read.csv("input/S4A_mod.csv",header=TRUE)
RF_O_data<-all[,c("mga2","lrp","gntR_spy0715","emm_type")]

set.seed(998)
inTraining <- createDataPartition(RF_O_data$emm_type, p = .65, list = FALSE)
training <- RF_O_data[ inTraining,]
testing  <- RF_O_data[-inTraining,]
trainX<-training[,1:3]
trainY<-training[,4]
testX<-testing[,1:3]
testY<-testing[,4]

CV_10_Folds <- createMultiFolds(training$emm_type, k = 10, times = 10)
fitControl <- trainControl(method = "repeatedcv", number = 10, repeats = 10, index=CV_10_Folds)

cl <- makeCluster(6, type = "SOCK")
registerDoSNOW(cl)
RF_O <- train(emm_type ~ ., data = training,
              method = "RRF",
              tuneLength = 3,
              ntree=1000,
```

```
                trControl = fitControl,
                flagReg=0,
                preProc=c("center","scale")
                )

predict_RF_O=predict(RF_O,testing)

stopCluster(cl)
RF_O

###############################################
# Regularised random forest: Cross validation
###############################################

all<-read.csv("input/S4A_mod.csv",header=TRUE)
RF_R_data<-all[,c("mga2","lrp","copY","crgR","emm_type")]

set.seed(998)
inTraining <- createDataPartition(RF_R_data$emm_type, p = .65, list = FALSE)
training <- RF_R_data[ inTraining,]
testing  <- RF_R_data[-inTraining,]

CV_10_Folds <- createMultiFolds(training$emm_type, k = 10, times = 10)
fitControl <- trainControl(method = "repeatedcv", number = 10, repeats = 10, index=CV_10_Folds)

cl <- makeCluster(6, type = "SOCK")
registerDoSNOW(cl)
RF_R <- train(emm_type ~ ., data = training,
                method = "RRF",
                tuneLength = 3,
                ntree=1000,
                trControl = fitControl,
```

```
                flagReg=1,
                preProc=c("center","scale")
                )

predict_RF_R=predict(RF_R,testing)

stopCluster(cl)
RF_R

##########################################
# Guided random forest: Cross validation
##########################################

all<-read.csv("input/S4A_mod.csv",header=TRUE)
RF_G_data<-
all[,c("mga2","lrp","spy1934","gntR_spy0715","rivR","M28_spy1337","spy1325","gntR_spy1602","sp
y1817","crgR","emm_type")]

myRRF <- list(label="Regularized Random Forest", type = c("Classification","Regression"),
            library = c("randomForest","RRF"),loop=NULL,tags=c("Random Forest","Ensemble
Model","Bagging","Implicit Feature Selection","Regularization"))
prm <- data.frame(parameter = c("mtry", "coefReg","coefImp"),
                class = rep("numeric", 3),
                label = c("Randomly Selected Predictors", "Regularization Value", "Importance
Coefficient"))
myRRF$parameters <- prm

RRFgrid <- function(x, y, len = NULL, search = "grid"){
  rf<-RRF(x,y,flagReg=0)
  GRF_coefReg<-(rf$importance/(max(rf$importance)))
  if(search == "grid") {
    out <- expand.grid(mtry = caret::var_seq(p = ncol(x),
                                classification = is.factor(y),
                                len = len),
```

```
                  #coefReg = seq(0.01, 1, length = len),
                  coefReg=GRF_coefReg,
                  coefImp = seq(0, 1, length = len))
  } else {
    out <- data.frame(mtry = sample(1:ncol(x), size = len, replace = TRUE),
                 #coefReg = runif(len, min = 0, max = 1),
                 coefReg=GRF_coefReg,
                 coefImp = runif(len, min = 0, max = 1))
  }
  out
}

myRRF$grid <- RRFgrid

RRFfit <- function(x, y, wts, param, lev, last, classProbs, ...) {
  theDots <- list(...)
  theDots$importance <- TRUE
  args <- list(x = x, y = y, mtry = param$mtry)
  args <- c(args, theDots)
  firstFit <- do.call(randomForest::randomForest, args)
  firstImp <- randomForest:::importance(firstFit)
  if(is.factor(y))
  {
    firstImp <- firstImp[,"MeanDecreaseGini"]/max(firstImp[,"MeanDecreaseGini"])
  } else
  firstImp <- firstImp[,"%IncMSE"]/max(firstImp[,"%IncMSE"])
  firstImp <- ((1 - param$coefImp) * param$coefReg) + (param$coefImp * firstImp)
  RRF::RRF(x, y, mtry = param$mtry, coefReg = firstImp, ...)
}

myRRF$fit <- RRFfit
```

```
RRFvarImp<-function(object, ...) {
  varImp <- RRF::importance(object, ...)
  if(object$type == "regression")
    varImp <- data.frame(Overall = varImp[,"%IncMSE"])
  else {
    retainNames <- levels(object$y)
    if(all(retainNames %in% colnames(varImp))) {
      varImp <- varImp[, retainNames]
    } else {
      varImp <- data.frame(Overall = varImp[,1])
    }
  }
  out <- as.data.frame(varImp, stringsAsFactors = TRUE)
  if(dim(out)[2] == 2) {
    tmp <- apply(out, 1, mean)
    out[,1] <- out[,2] <- tmp
  }
  out
}

myRRF$varImp<-RRFvarImp

RRFpred <- function(modelFit, newdata, submodels = NULL)
  predict(modelFit, newdata)

myRRF$predict <- RRFpred

RRFprob <- function(modelFit, newdata, submodels = NULL)
  predict(modelFit, newdata, type = "prob")

myRRF$prob <- RRFprob
```

```
myRRF$sort <- function(x) x[order(x$coefReg),]

myRRF$levels <- function(x) x$obsLevels

set.seed(998)
inTraining <- createDataPartition(RF_G_data$emm_type, p = .65, list = FALSE)
training <- RF_G_data[ inTraining,]
testing  <- RF_G_data[-inTraining,]

CV_10_Folds <- createMultiFolds(training$emm_type, k = 10, times = 10)
fitControl <- trainControl(method = "repeatedcv", number = 10, repeats = 10, index=CV_10_Folds)

set.seed(825)
cl <- makeCluster(6, type = "SOCK")
registerDoSNOW(cl)
RF_G <- train(emm_type ~ ., data = training,
              method = myRRF,
              tuneLength = 3,
              ntree=1000,
              trControl = fitControl,
              flagReg=0,
              preProc=c("center","scale")
              )

stopCluster(cl)
RF_G

##############################################
# Ordinary random forest: Performance metrics
##############################################

library(pROC)
```

```
library(caret)
library(doSNOW)
library (RRF)

all<-read.csv("input/S4A_mod.csv",header=TRUE)
RF_O_ROC_data<-all[,c("mga2","lrp","gntR_spy0715","emm_type")]

inTraining <- createDataPartition(RF_O_ROC_data$emm_type, p = .65, list = FALSE)
training <- RF_O_ROC_data[ inTraining,]
testing  <- RF_O_ROC_data[-inTraining,]
train<-RF_O_ROC_data[1:629,]
trainX<-train[,c("mga2","lrp","gntR_spy0715")]
trainY<-train[,"emm_type"]
test<-RF_O_ROC_data[630:944,]
testX<-test[,c("mga2","lrp","gntR_spy0715")]
testY<-test[,"emm_type"]

set.seed(998)
RF_O_ROC<-RRF(trainX,as.factor(trainY),flagReg=0)

RF_O_ROC_predict<-predict(RF_O_ROC,testX,type="vote")

cl <- makeCluster(6, type = "SOCK")
registerDoSNOW(cl)

multiclass.roc(as.factor(testY),RF_O_ROC_predict)

stopCluster(cl)

RF_O_CM_predict<-predict(RF_O_ROC,testX)
RF_O_CM <- confusionMatrix(RF_O_CM_predict, reference = as.factor(testY))
RF_O_CM
```

```
# # extract F1 score for all classes
RF_O_CM[["byClass"]][ , "F1"] #for multiclass classification problems

################################################
# Regularized random forest: Performance metrics
################################################


library(pROC)
library(caret)
library(doSNOW)
library (RRF)

all<-read.csv("input/S4A_mod.csv",header=TRUE)
RF_R_ROC_data<-all[,c("mga2","lrp","copY","crgR","emm_type")]

inTraining <- createDataPartition(RF_R_ROC_data$emm_type, p = .65, list = FALSE)
training <- RF_R_ROC_data[ inTraining,]
testing  <- RF_R_ROC_data[-inTraining,]
train<-RF_R_ROC_data[1:629,]
trainX<-train[,c("mga2","lrp","copY","crgR")]
trainY<-train[,"emm_type"]
test<-RF_R_ROC_data[630:944,]
testX<-test[,c("mga2","lrp","copY","crgR")]
testY<-test[,"emm_type"]

set.seed(998)
RF_R_ROC<-RRF(trainX,as.factor(trainY),flagReg=1)

RF_R_ROC_predict<-predict(RF_R_ROC,testX,type="vote")

cl <- makeCluster(6, type = "SOCK")
```

```
registerDoSNOW(cl)

multiclass.roc(as.factor(testY),RF_R_ROC_predict)

stopCluster(cl)

RF_R_CM_predict<-predict(RF_R_ROC,testX)
RF_R_CM <- confusionMatrix(RF_R_CM_predict, reference = as.factor(testY))
RF_R_CM
# # extract F1 score for all classes
RF_R_CM[["byClass"]][ , "F1"] #for multiclass classification problems

############################################
# Guided random forest: Performance metrics
############################################


library(pROC)
library(caret)
library(doSNOW)
library (RRF)

all<-read.csv("input/S4A_mod.csv",header=TRUE)
RF_G_data<-
all[,c("mga2","lrp","spy1934","gntR_spy0715","rivR","M28_spy1337","spy1325","gntR_spy1602","sp
y1817","crgR","emm_type")]

train<-RF_G_data[1:629,]
trainX<-
train[,c("mga2","lrp","spy1934","gntR_spy0715","rivR","M28_spy1337","spy1325","gntR_spy1602",
"spy1817","crgR")]
trainY<-train[,"emm_type"]

test<-RF_G_data[630:944,]
```

```
testX<-
test[,c("mga2","lrp","spy1934","gntR_spy0715","rivR","M28_spy1337","spy1325","gntR_spy1602","
spy1817","crgR")]

testY<-test[,"emm_type"]


set.seed(998)


rf<-RRF(trainX,trainY,flagReg=0)

GRF_coefReg<-(rf$importance/(max(rf$importance)))

RF_G_ROC<-RRF(trainX,as.factor(trainY),flagReg=0,coefReg=GRF_coefReg)


RF_G_ROC_predict<-predict(RF_G_ROC,testX,type="vote")


cl <- makeCluster(6, type = "SOCK")

registerDoSNOW(cl)


multiclass.roc(as.factor(testY),RF_G_ROC_predict,plot=TRUE)


stopCluster(cl)


RF_G_CM_predict<-predict(RF_G_ROC,testX)

RF_G_CM <- confusionMatrix(RF_G_CM_predict, reference = as.factor(testY))

RF_G_CM

# # extract F1 score for all classes

RF_G_CM[["byClass"]][ , "F1"] #for multiclass classification problems
```
